# What microRNAs could tell us about the human X chromosome

**DOI:** 10.1007/s00018-020-03526-7

**Published:** 2020-04-30

**Authors:** Armando Di Palo, Chiara Siniscalchi, Mariacarolina Salerno, Aniello Russo, Claus Højbjerg Gravholt, Nicoletta Potenza

**Affiliations:** 1 Department of Environmental, Biological and Pharmaceutical Sciences and Technologies, University of Campania “Luigi Vanvitelli”, Caserta, Italy; 2 Pediatric Endocrine Unit, Department of Translational Medical Sciences, University of Naples Federico II, Naples, Italy; 3 Department of Endocrinology and Internal Medicine, Aarhus University Hospital, Aarhus, Denmark; 4 Department of Molecular Medicine, Aarhus University Hospital, Aarhus, Denmark

**Keywords:** microRNA, X chromosome, Turner syndrome, Klinefelter syndrome

## Abstract

MicroRNAs (miRNA) are small-non coding RNAs endowed with great regulatory power, thus playing key roles not only in almost all physiological pathways, but also in the pathogenesis of several diseases. Surprisingly, genomic distribution analysis revealed the highest density of miRNA sequences on the X chromosome; this evolutionary conserved mammalian feature equips females with a larger miRNA machinery than males. However, miRNAs contribution to some X-related conditions, properties or functions is still poorly explored. With the aim to support and focus research in the field, this review analyzes the literature and databases about X-linked miRNAs, trying to understand how miRNAs could contribute to emerging gender-biased functions and pathological mechanisms, such as immunity and cancer. A fine map of miRNA sequences on the X chromosome is reported, and their known functions are discussed; in addition, bioinformatics functional analyses of the whole X-linked miRNA targetome (predicted and validated) were performed. The emerging scenario points to different gaps in the knowledge that should be filled with future experimental investigations, also in terms of possible implications and pathological perspectives for X chromosome aneuploidy syndromes, such as Turner and Klinefelter syndromes.

**Electronic supplementary material:**

The online version of this article (10.1007/s00018-020-03526-7) contains supplementary material, which is available to authorized users.

## Introduction

MicroRNAs (miRNA) are small non-coding RNAs that post-transcriptionally regulate gene expression by affecting both translation and stability of complementary mRNAs [1]. Bioinformatics predictions indicate that mammalian miRNAs regulate 30–50% of all protein-coding genes; each miRNA can bind several mRNAs and each mRNA can be targeted by different miRNAs, thus giving rise to complex regulatory networks that take part in the regulation of almost all physiological pathways [2, 3]. As a consequence, miRNA mutations, dysregulation of their expression or dysfunction of miRNA biogenesis have a key role in different pathological processes, with oncogenesis as the most investigated field [4–6].

miRNA genes can be present singularly in the genome or clustered together, with their own promoter or hosted by coding or non-coding genes, thus generally sharing their transcription promoter [7]. They are transcribed by RNA polymerase II as long primary transcripts (pri-miRNAs), wherein miRNA sequences fold into hairpin structures, recognized and excised by Drosha and DGCR8, the microprocessor complex, generating 60–80 nt precursors (pre-miRNAs). Pre-miRNAs are exported to the cytoplasm where they are processed by Dicer in miRNA duplexes. Finally, the mature miRNA strand that could derive from the 5′ arm or 3′ arm of the pre-miRNA (miRNA-5p or miRNA-3p, respectively) is loaded onto an Argonaute protein within the RISC complex to bind and silence complementary mRNA targets [8].

Surprisingly, genomic distribution analysis revealed that the human X chromosome has a higher density of miRNAs when compared to autosomes [9]; in contrast, the Y chromosome has only 4 miRNA sequences, but not experimentally validated, with 2 produced by the PAR1 (pseudoautosomal region 1) shared by both sex chromosomes. The miRNA enrichment on the X chromosome was observed in several mammalian species, but does not extend to species other than mammals [9]. The evolutionary conserved higher density of miRNAs on the X chromosome may suggest that X-linked miRNAs could contribute to some X-related conditions, properties or functions in mammals.

It has long been known that the presence of two X chromosomes in females and only one X chromosome in males requires mechanisms to equalize gene dosage between sexes and relative to autosomes to avoid a potentially lethal double-dose of genes residing on the X chromosome [10]. This mechanism involves two processes: X chromosome inactivation (XCI), i.e., the silencing of almost all genes on the one X chromosome leading to partial functional X monosomy, and X chromosome upregulation leading to increased gene expression on the single active X chromosome in males or females. It has been shown that up to 15% of the X-linked genes escape permanent silencing (“escapees”), with large variability in their number and tissue distribution within a given individual and between individuals: the escape from silencing or skewed XCI allow the expression of some genes by both X chromosomes in females [11, 12]. Furthermore, during early female embryonic development, the process of XCI is random across alleles in all cells, i.e., occurs irrespective of the parental origin of X chromosome and is clonally maintained once established, thus resulting in females being a functional mosaic for the active X chromosome across cell types. The XCI is a multistep process, involving a mechanism of counting and choice of the chromosome that will start XCI. The entire course of action is directed by the X inactivation center (Xic), a nuclear complex constituted by many non-coding DNA elements and genes (XIST, etc.) that lead to packaging into transcriptional inactive heterochromatin [13], also known as the Barr body.

X chromosome aneuploidies interfering with dosage compensation cause widely variable symptoms in two different syndromes, in particular Turner syndrome (with the classical karyotype 45,X), caused by a partial or complete lack of a second X chromosome in females, and Klinefelter syndrome (with the classical karyotype 47,XXY), caused by two or more X chromosomes in males. In both syndromes no obvious genotype–phenotype relationship has so far been established, and patients carrying the same karyotype may exhibit widely differing traits, suggesting a role of epigenetic mechanisms behind sex chromosome aneuploidy [14, 15]. So far, some studies have been dedicated to this issue, essentially based on the analysis of epigenetic mechanisms, as DNA methylation and histone modifications [16, 17], and recently a dose effect for the number of X chromosomes has been shown to affect the expression profile for a range of mRNAs in a comparison of 45,X, 46,XX, 46,XY and 47,XXY individuals [18], implying that a similar dose effect could be present for X-linked miRNAs. But hitherto, the contribution of miRNAs to the phenotype of these syndromes has very poorly investigated [19–21], even though the higher density of miRNAs mapped on the X chromosome and their recognized regulatory role in biological processes.

In this review, we analyze the literature and databases about X-linked miRNAs, aiming at understanding how miRNAs could contribute to emerging gender-biased functions and to highlight some gaps in the knowledge, particularly in terms of possible implications and pathological perspectives for X chromosome aneuploidy syndromes.

## Mapping of miRNA sequences on X chromosome

### A general overview

**Fig. 1:**
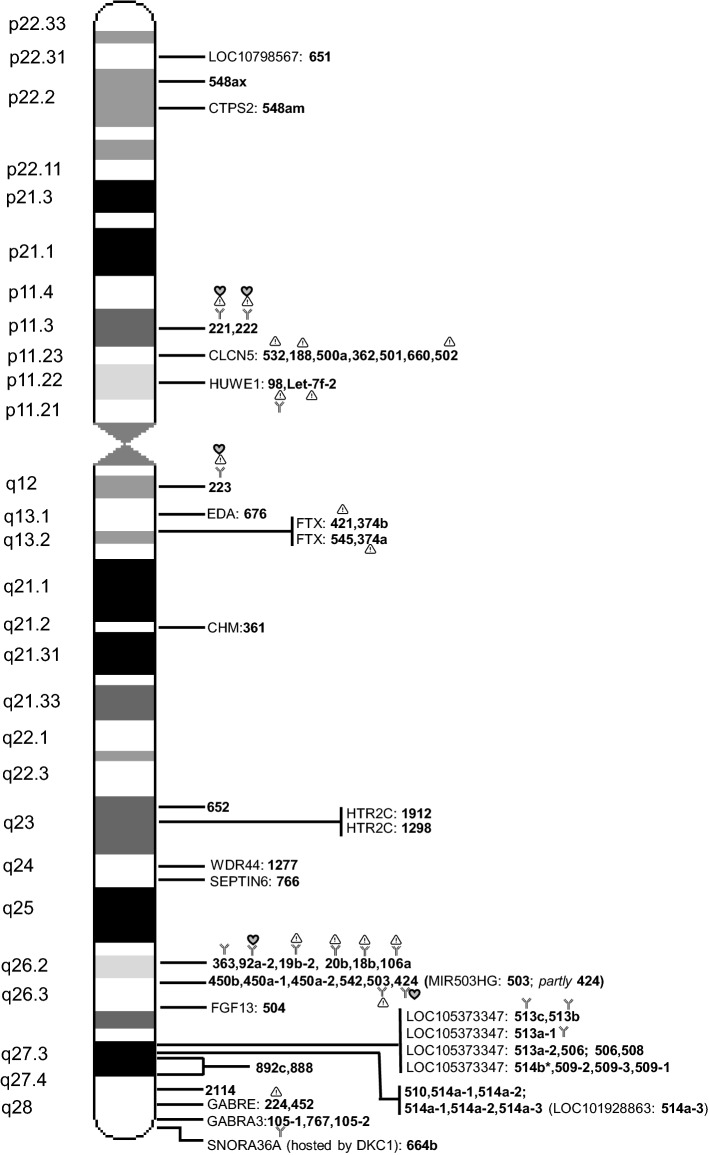
Map of microRNA sequences on human X chromosome. Information about validated miRNA sequence position is based on Ensembl and miRBase databases. On the left, chromosome bands are indicated and on the right, the miRNA names, simplified by eliminating « hsa-miR- » and indicated only by the number. Some miRNAs are intragenic and the host gene is indicated (name of host gene). Several miRNAs are clustered (within 10 kbp) and are shown on the same line separated by ‘,’, with the exception of miR-514b (*) that is located within 10.3 kbp of adjacent cluster; miRNA name is repeated when it can be considered belonging to different clusters. Some miRNAs are mainly involved in immunity ( ), cancer ( ) and cardiovascular homeostasis ( ), as detailed in the text. *LOC10798567*, uncharacterized non-coding RNA; *CTPS2*, CTP synthase 2; *CLCN5*, chloride voltage-gated channel 5; *HUWE1*, HECT, UBA and WWE domain containing E3 ubiquitin protein ligase 1; *EDA*, ectodysplasin A; *FTX*, FTX transcript, XIST regulator; *CHM*, CHM Rab escort protein; *HTR2C*, 5-hydroxytryptamine receptor 2C; *WDR44*, WD repeat domain 44; *SEPTIN6*, septin 6; *MIR503HG*, miR-503 host gene; *FGF13*, fibroblast growth factor 13; *LOC105373347*, periphilin-1-like; *LOC101928863*, uncharacterized non-coding RNA; *GABRE*, gamma-aminobutyric acid type A receptor epsilon subunit; *GABRA3*, gamma-aminobutyric acid type A receptor alpha3 subunit; *SNORA36A*, smal nucleolar RNA, *H/ACA box 36A*; DKC, dyskerin pseudouridine synthase 1. The figure was inspired by Pinheiro et al. (2011) [35]

The human X chromosome contains 10% of all miRNAs detected so far in the human genome. According to the miRbase database, 118 miRNAs are located on the X chromosome, with 62 classified as “with confidence”, i.e., experimentally validated. Exploring Ensembl and miRbase databases (https://www.ensembl.org/Homo_sapiens/; https://www.miRBase.org), positions of the miRNA sequences were mapped on the X chromosome as well as their genomic context, i.e., their possible position in host gene and transcribed strand. The full miRNA list is available as Supplementary Material, whereas some statistical parameters are reported in Table 1: approximately 70% of miRNA sequences are localized on Xq, the phylogenetically oldest part of the X chromosome [22]; more than the half (62.7%) are intragenic, suggesting a co-regulation of miRNAs and host genes, in particular when they are transcribed by the same strand of host gene, and indeed this is the most often the case. We focused our attention on the 62 miRNAs experimentally validated and they are reported in Fig. 1; most of them are clustered (77.3%), and hosted in either coding or non-coding genes (66%).

**Table 1:** Information about miRNA sequence mapping on X chromosome, based on Ensembl and miRBase databases

miRNAs	Xp	Xq	Strand	Intragenic (same strand of host gene)	Intergenic
62 “Confidence”	14	48	16 (+), 46 (−)	41 (31)	21
56 “Non confidence”	22	34	25 (+), 31 (−)	33 (24)	23
118 Total	36	82	41 (+), 77 (−)	74 (55)	44

### Genes related to X-linked syndromes: any relationships with hosted miRNAs?

Some of the hosting coding genes are directly involved in X-linked syndromes. This is the case of *HUWE1*, an E3 ubiquitin ligase required for the ubiquitination and subsequent degradation of different targets, such as the anti-apoptotic protein Mcl1 [myeloid cell leukemia sequence 1 (BCL2-related)], the tumor suppressor p53, core histones and DNA polymerase beta; mutations in the HUWE1 gene are associated with X-linked syndromic cognitive disability, where researchers found copy number variations [23], that perhaps could have similarities with some of the neurocognitive challenges seen in some individuals with Turner syndrome. It has also been proposed that an increased propensity to harbor copy number variations should be present in Turner syndrome (45,X) [24], while no studies so far have investigated this in the other sex chromosome aneuploidy syndromes. Defects in the *EDA* gene, hosting miR-676 and encoding a type II membrane protein belonging to the tumor necrosis factor family, are a cause of ectodermal dysplasia, anhidrotic, which is also known as X-linked hypohidrotic ectodermal dysplasia [25]. Another hosting miRNA gene, *CHM*, has been involved in an X-linked disease, choroideremia, also known as tapetochoroidal dystrophy, characterized by progressive dystrophy of the choroid, retinal pigment epithelium and retina [26]. Possible miRNA contribution to all those syndromes involving X-linked genes hosting miRNAs has not been explored.

Noteworthy, two different miRNA clusters are also hosted (and likely co-regulated) by the *FTX* gene, located upstream of *XIST* within the X-inactivation center (XIC), and producing a long non-coding RNA endowed with a central role in positively regulating the expression of *XIST*, essential for the initiation and spread of X-inactivation [27]. A relationship between *FTX* and miR-374a has been reported: *FTX* sponges miR-374a, resulting in the repression of Wnt/β-catenin signaling activity and consequent inhibition of hepatocellular carcinoma cell (HCC) epithelial–mesenchymal transition and invasion; moreover, *FTX* inhibits proliferation of HCC cells by binding the DNA replication licensing factor MCM2, and it is significantly downregulated in HCC tissues compared with normal liver tissues. These features point to a tumor suppressor role for *FTX*: together with the notion that *FTX* is expressed at higher levels in female in comparison to male livers, it could be speculated that the described mechanisms contribute to the higher susceptibility of males than females to HCC [28].

Finally, although a precise map of XCI escapee miRNAs is still missing, some miRNAs are located within genes that have been shown to escape XCI, i.e., *CTPS2*, *HTR2C*, *GABRE* [29], *FTX* [27], suggesting that also the hosted miRNAs could escape silencing. It is known that with female mosaicism, i.e., random inactivation of one of the two parental X chromosome and clonal transmission to the daughter cells, silencing escapees or skewed patterns of inactivation of X genes could lead to unbalanced expression between sexes and to sex-specific responses; however, miRNA contribution to these phenomena has not been explored, even though the existence of multiple targets for each miRNA could result in a cascade-like effect, leading to greater gender differences in terms of gene regulation.

## Role of X-linked miRNAs emerging in the literature

### Immunity and cancer

Based on literature data, the two emerging (or most studied) pathways involving regulation by X-linked miRNAs are immunity and cancer, two processes strictly linked with each other.

A significant difference between the male and female immune system function has been described: from childhood to old age, the female immune system appears more flexible, appropriate and able to better counteract infections and non-infectious disease, including cancer; females have longer life expectancies and an improved survival outcome from shock episodes caused by sepsis, injury or trauma-hemorrhage. However, this advantage can become detrimental, since the sex-biased nature of the immune system can result in an increased susceptibility to develop autoimmune diseases [30, 31]. Gender-specific immune responses may be partially explained by hormonal regulation; however, the importance of the X chromosome in shaping immune functions and autoimmunity has been recognized, since the presence of several X-located genes with a direct or an indirect role in immunity, including several members of the Toll-like receptor family pathway, with some of them being responsible for X-linked primary immunodeficiency (PID) [32–34]. A number of X-linked miRNAs impacting the immune system integrity and function potentiate the concept of the immunological advantage of females [reviewed in 35]. Among the X-linked miRNAs involved in immune regulation, miR-223 is probably the most studied so far. miR-223, which is also involved in cancer pathology, is expressed in the myeloid lineage in the bone marrow, and is a regulator of neutrophil differentiation from myeloid precursors; it is also a negative modulator of the inflammatory response [36, 37]. Other miRNAs mapping on X chromosome are involved in hematopoietic lineage differentiation. miR-106 was shown to negatively control monocytopoiesis by targeting *AML*-*1*; intriguingly, all miRNAs of the miR-106–363 cluster have oncogenic potential and been implicated in human T cell leukemias, where they are overexpressed [38]. In addition, two clustered miRNAs, miR-424 and miR-503, together with miR-222, all X-linked, promote monocytic differentiation [39]. The involvement of X-linked miRNAs in gender-biased immunity is also supported by a study reporting differential expression of six X-linked miRNAs (miR-221, miR-222, miR-98, miR-532, miR-106 and miR-92a) in PBMC (peripheral blood mononuclear cells) between males and females affected by rheumatoid arthritis, an autoimmune disease that affects females three times more often than men [40].

An example of a miRNA-dependent, sex-specific regulation of immune responses and cancer immunosurveillance is represented by the PD-1/PD-L1 pathway. The PD1 receptor, by binding to PD-L1 (or PD-L2), induces T cells to undergo immunosurveillance against tumors; PD-1 is also considered an immune checkpoint key molecule. Intriguingly, a number of X-linked miRNAs directly targets the stability and/or the translation of PD-L1 transcript (i.e., miR-20a/b, miR-106 a/b, miR-513, miR-424), or interfere with the transcription factors modulating its expression, i.e., miR-18a/b targeting HIF and miR-221/222 targeting STAT3 [31].

Several forms of cancer show a gender difference in terms of incidence, prevalence, and response to therapy [41]. miR-221 and miR-222 are the most extensively studied in tumors of different origins, wherein they act as oncomirs (oncogenic miRNAs) controlling the development and progression of the tumor through the down-regulation of several key targets [42, 43]. Both miRNAs are located in a cluster on the X chromosome; their deregulation is a hallmark of several types of cancer, probably because one of their targets is the cell cycle regulatory protein p27Kip1/CDKN1B [44]. In contrast to miR-221 and miR-222, different studies point to a tumor suppressor role for miR-503: in different cancer cell lines, it has been shown to target modulators of the cell cycle, such as cyclin-dependent kinases (CCND1, D2, D3) and cyclins (D1 and D3); consistently, miR-503 was found down-regulated in cancer tissues [reviewed in 45]. However, a possible differential contribution of miR-503 to tumors between males and females can presently not be evaluated since the gender context was ignored in current studies. This consideration is applicable also to other X-linked miRNAs implicated in different types of cancer [35].

With regard to breast cancer, the most common cancer in women, two profiling studies, performed on very large patient cohorts, indicate that circulating miRNAs from the two X-linked miR-106a–363 and miR-532–502 clusters are promising non-invasive biomarkers for diagnosis since they were upregulated in patients in comparison to healthy volunteers [46, 47]. Functional studies based on cell lines experiments showed an implication of some of those miRNA in breast cancer; in particular, miR-532, miR-19b and miR-20b act as oncomirs by down-regulating the tumor suppressors *RERG* (ras-related and estrogen-regulated growth inhibitor), *PTPRG* (protein tyrosine phosphatase receptor type G) and *PTEN* (phosphatase and tensin homologue), respectively [48–50]; in contrast, miR-188 and miR-502 act as tumor suppressors by inhibiting *TMED3* (Transmembrane Emp24 Protein Transport Domain Containing 3) and the H4K20 methyltransferase *SET8*, respectively [51, 52]. Overall, further functional studies are required to put together the different pieces of the puzzle to gain a comprehensive view of possible X-linked miRNAs contribution to breast cancer.

### Cardiovascular homeostasis

In the cardiovascular system, regulatory power of microRNAs has been invoked at multiple level for controlling functions of various cells, such as cardiomyocytes, endothelial cells, smooth muscle cells, and fibroblasts [53]. Intriguingly, more women than man die of cardiovascular disease every year [54]. Based on the notion that estrogen can regulate transcription and miRNA processing, and that some X-linked miRNAs could escape XCI, it can be argued that miRNAs contribute to some sex-biased features of cardiovascular homeostasis [55]. Most of the studies into the field are based on profiling analyses of cellular and circulating miRNAs, with some insights into molecular mechanisms acquired from murine or rat models, whose extrapolation for humans should be viewed with caution. However, for some X-linked miRNAs, an association with biological pathways involved in cardiovascular homeostasis has been accomplished, although using human endothelial cells and thus requiring further validation in human subjects. For instance, miR-221/-222, in addition to their well-known role in cancer, are probably involved also in vascular and cardiac diseases since they can indirectly reduce the expression of endothelial nitric oxide synthase (eNOS), and NO impairment is a feature of different cardiomyopathies [53]; moreover, gender expression disparities have been reported, with decreased expression in at least murine hearts of miR-222, leading to higher levels of eNOS [56]. miR-92a and miR-223 inhibit angiogenesis by targeting integrin subunit alfa 5 and integrin beta 1, respectively [57, 58]. In contrast, miR-424 promote angiogenesis in human endothelial cells subjected to hypoxia by targeting cullin-2, a scaffolding protein critical to the assembly of the ubiquitin ligase system, thereby stabilizing the transcription factor hypoxia-inducible factor 1α (HIF-1α), which regulates genes governing angiogenesis and metabolic pathways [59].

### Male fertility

A biological pathway possibly regulated by X-linked miRNAs that deserves investigation in the near future could be male fertility. In fact, it is recognized that the X chromosome is also correlated to male fertility, because of the presence of a relatively high number of testis-specific genes [60, 61]. This observation is probably applicable also to miRNAs: Guo et al. demonstrated that the highest proportion of X-linked miRNAs was found in mouse testis; moreover, comparison between human and mouse showed that they had an average substitution rate higher than other testis miRNAs on autosomes, implicating an miRNA contribution in male reproductive function and mammalian speciation [9, 62]. Supporting this hypothesis, one group of X-linked miRNAs was identified as a very quickly evolving miRNA cluster because of its presence restricted to primates; they are all hosted in the uncharacterized non-coding RNA (LOC105373347) (Fig. 1), and preferentially expressed in testicular tissues [63–65]. Functional investigations of the role of these miRNAs in male fertility are still missing. One miRNA from the cluster, miR-513, was shown to be able to downregulate the expression of the receptor for luteinizing hormone (LH) and human chorionic gonadotropin (hCG), two closely related hormones crucial not only for male but also female reproductive processes; however, the study was performed on primary granulosa cell cultures, thus requiring further validation in human subjects [66].

## Targetome of X-linked miRNAs and its functional analysis using bioinformatics

### Targetome and biological pathways

To possibly achieve new perspectives on the biological role of X-linked miRNAs, we decided to analyze their entire targetome and related biological pathways.

miRNAs exert their biological role by down-regulating specific targets; given that binding of miRNA to mRNA tolerates mismatches, except for the seed sequence (nucleotides 2–8 of the miRNA) requiring a perfect complementarity, each miRNA can potentially bind hundreds of mRNAs. Thus, identifying the entire “true” miRNA targetome is still challenging, even though highly informative concerning the role of miRNA in health and disease. Generally, the approach is based on a first step of bioinformatics prediction, followed by an experimental validation of those targets predicted by more than one bioinformatics tool. For our analyses, we considered 80 miRNAs directly related to the group of the 62 validated miRNAs, since some pre-miRNAs yield both -5p and -3p isoforms, experimentally validated, and in addition different pre-miRNAs produce the same mature miRNA (Supplementary Material). Exploring the miRWalk platform [67], for each miRNA we considered all the experimentally validated targets reported by the miRTarbase tool and predicted targets identified by both TargetScan and miRDB tools, with a cut-off score ≥ 0.5 for miRNA-mRNA pairings, in both UTRs and coding sequence of target transcripts. This approach produced a list of 6675 non-redundant transcripts, representing the targetome of X-linked miRNAs (Supplementary Material).

**Fig. 2:**
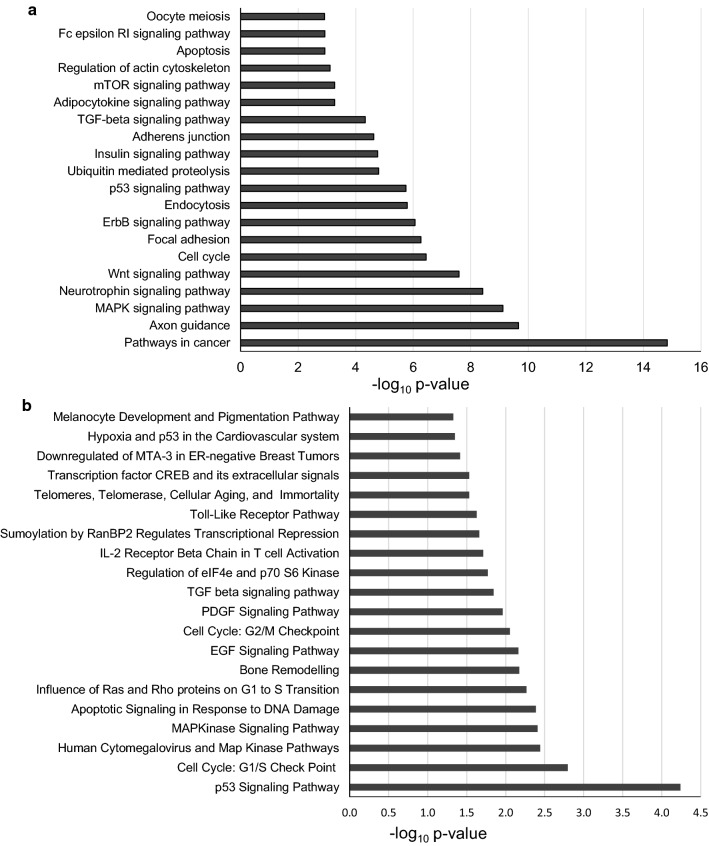
Biological pathways potentially governed by X-linked microRNAs. The whole targetome (predicted and validated) of X-linked microRNAs was analyzed by the program DAVID. **a** Top-20-*p* values significantly enriched pathways according to KEGG (full list as Supplementary Material) or **b** Biocarta. Biological pathways were considered statistically significant if *p* value was less than 0.05 (Benjamini–Hochberg procedure for multiple correction)

Pathway enrichment analysis of target genes was performed using the program DAVID (https://david.ncifcrf.gov/) that consists of an integrated biological knowledgebase and analytic tools aimed at systematically extracting biological meaning from large gene/protein lists [68]. In particular, we focused on the significantly enriched pathways using KEGG (Fig. 2a and Supplementary Material) and Biocarta (Fig. 2b) as reference databases.

We found different pathways predicted by both tools, such as “p53 signaling pathway”, “MAPK pathways” and particularly “Cell cycle” and its checkpoints. This last observation was already reported for enriched terms in the targetome of testis X-linked miRNAs and was put in relation to massive and continuous cell division during spermatogenesis [9]. Different pathways were also involved in immunity, and probably this is an expected result considering the data emerging from the literature and discussed in the previous paragraphs. KEGG pathways point also to involvement of the X-linked miRNA targetome in “Axon guidance” and “Neurotrophin pathway” (the second and the fourth most significantly enriched pathways, respectively); those fields are absolutely unexplored in terms of miRNA contribution, even though the contribution of genes on the X-chromosome to the nervous system development and functioning is known, as well as genes associated with X-linked intellectual disability (XLID) [69] and some X-linked miRNAs predicted to be involved in X-linked and autosomal intellectual disability (ID) gene regulation [70]. For example, *HUWE1* is a gene implicated in XLID [71], and it hosts an miRNA cluster, whose possible contribution to the disease has not been explored yet.

The most significantly enriched pathway indicated by KEGG is “Pathways in cancer”: this is probably not surprising, as microRNAs role in carcinogenesis represents one of the hot topic in contemporary research. However, specific contributions of X-linked miRNAs and/or gender context still need further study.

Finally, among the top-20-*p* value scoring of enriched pathways from KEGG emerges “Oocyte meiosis” and in the full list (Supplementary material) also “Progesterone-mediated oocyte maturation”: this field should be explored in the near future, also in terms of possible contribution to ovarian failure observed in Turner syndrome patients.

### Methods

The list of 80 X-linked miRNAs was used to launch a search on miRWalk platform (http://mirwalk.umm.uni-heidelberg.de/) to retrieve all their target genes. In detail, by selecting “miRTarbase”, or “TargetScan” and “miRDB”, and “5′ UTR”, or “3′ UTR” or “coding region” and selecting the lower score (0.5) for miRNA–mRNA pairings, 6 different target lists were retrieved containing all experimentally validated targets (by miRTarbase tool), or those predicted by both TargetScan and miRDB tools. By exporting the lists in Excel, a unique list of 6675 non-redundant transcripts representing the whole targetome of X-linked miRNAs was produced. On that list, enrichment of biological pathways supplied by KEGG and Biocarta was performed by Database for Annotation, Visualization and Integrated Discovery (DAVID, http://david.abcc.ncifcrf.gov/), which facilitates the transition from data collection to biological analysis and provides a comprehensive set of functional annotation tools to condense large gene lists into gene functional groups, to convert between gene/protein identifiers, to visualize many-gene-to-many-term relationships, to cluster redundant and heterogeneous terms into groups, to search for interesting and related genes or terms, and, dynamically, to view genes from their lists on bio-pathways. In detail, the pathway enrichment on our list of transcripts was quantitatively measured by statistical methods, including Chi-square, Fisher’s exact test, binomial probability, and hypergeometric distribution. Considering this type of analysis could increase the false-positive rate, to control the false-positive rate in the results, a multiple test correction of enrichment p values was performed on the functional annotation categories by selecting the Benjamini–Hochberg procedure. Biological pathways with *p* values < 0.05 were considered statistically significant [72].

## microRNAs in the genotype–phenotype relationship of Turner and Klinefelter syndrome patients

The partial or complete lack of a second X chromosome in a female causes Turner syndrome, estimated 1 in 2000 females [73]. 40–50% of Turner Syndrome women have the 45,X karyotype, 15–25% have mosaicism with 45,X/46,XX and about 3% present with 45,X/46,XY. 20% of cases have alterations of the X chromosome, with isochromosome Xq being the most frequent; ring X chromosome with deletion of either Xp or Xq are also present and 10–12% of cases have differing amounts of Y chromosome material. Phenotypic traits include short stature, infertility, cardiac malformations, bone dysgenesis, neurocognitive challenges, and autoimmune diseases, but also some external features such as short neck with webbing, and lymphedema; however, the external appearance and internal anomalies of Turner syndrome individuals vary greatly and cannot be predicted based on the karyotype—thus, there does not seem to be a clear karyotype–phenotype relation. When diagnosed in the childhood, Turner syndrome patients are offered therapy with growth hormone aimed at increasing adult height and later with sex hormone replacement therapy, due to gonadal dysgenesis resulting in hypergonadotropic hypogonadism [74].

It has become evident that Turner syndrome is much more than simple genetics, as no obvious karyotype–phenotype relationship exists: two individuals with the exact same karyotypes, can exhibit very different traits and comorbidities. The relationship between karyotype and phenotype is certainly complicated by the mosaicism (presence of more than one cell lines with X aneuploidies), that varies with tissue type and age of a given person, but also suggests a role for epigenetics mechanisms [75]. So far, few studies have been dedicated to that issue, essentially based on analysis of DNA methylation and histone modifications [16]. Regarding miRNAs, one paper unveils the role of miR-320a, found upregulated in PBMC and fetal gonadal tissues of 45,X TS females, and thus causing downregulation of its target KITLG, essential for ovarian development and primordial cell survival. This mechanism could partially account for the Turner syndrome-associated gonadal dysgenesis. miR-320 is not an X-linked miRNA, but its expression is modulated by *KDM5C*, encoded by an XCI-escaping gene [18], which is also differentially expressed in Turner syndrome [14]. Furthermore, a number of circulating miRNAs were differentially detected in 45,X Turner syndrome females as compared with 46,XX normal women, whose role should be further clarified [19].

Presence of an extra X-chromosome characterizes Klinefelter syndrome males, 1 in every 660 male births. Klinefelter syndrome is characterized by a 47,XXY karyotype in about 80–90% of men, whereas the remaining cases are represented by mosaicism, additional sex chromosome (e.g., 48,XXXY; 48,XXYY; 49,XXXXY) or X chromosome structural abnormalities. The low testosterone levels, hypergonadotropic hypogonadism observed in a high proportion of these men only explain a few of the classical characteristics of Klinefelter syndrome, such as tall stature, gynecomastia, while the frequent infertility encountered is a consequence of the dramatic demise of the seminiferous tubules which takes place early in life [76]. Also in the case of Klinefelter syndrome, the wide variable phenotypic spectrum and the different severity of symptoms suggest a role for epigenetic factors. Two different studies have profiled miRNAs in peripheral blood cells from a small group of non-mosaic Klinefelter syndrome patients and healthy men and found 89 [20] or 2 [21] miRNAs differentially detectable. Also in the case of Klinefelter syndrome, the functional contribution of miRNAs to the phenotype has not been explored.

Potential key X-linked genes responsible for the comorbidities seen in Turner syndrome, Klinefelter syndrome and other X chromosome aneuploidy syndromes have been identified. One example is represented by *KDM6A*, involved in germ cell development, differentially expressed, and methylated in both syndromes [14]; another example is *KDM5C*, which could play a role in the neurocognitive development of the syndromes [77]; *TIMP1*/*TIMP3* genes are involved in the bicuspid aortic valve, probably accounting for higher incidence of aortic dissection in Turner syndrome [78]. The *SHOX* gene (short stature homeobox), located on Xp22.23, is the only X-chromosome gene that is truly linked with a phenotypic trait in Turner syndrome, i.e., short stature and skeletal growth [79]. The function is dosage dependent, thus causing short stature in Turner syndrome and increased stature in other X aneuploidy condition characterized by extra X chromosomes (47,XXX; 47,XXY; 48,XXYY) [80].

With regard to miRNAs, currently only speculations are possible. For example, taking into account the number of X-linked miRNAs with a role in immunity, it is paradigmatic that a plethora of autoimmunity stigmata are observed in Turner syndrome patients [81]; consistently, Klinefelter syndrome males (47,XXY) have a risk similar to female to develop not only systemic lupus erythematosus (SLE), an autoimmune disease with a striking female preponderance [82], but also other autoimmune conditions [83] and as such both missing an X chromosome (like in Turner syndrome) and having too many (like in Klinefelter syndrome) can lead to an increased risk of autoimmune disease. The number of X chromosomes, and the consequent altered X-linked genes and miRNAs dosage, is probably critical for the maintenance or the loss of the immune homeostasis. Again, a role for X-linked miRNA in infertility of Klinefelter syndrome men could be hypothesized, based on some data in the literature discussed above: miRNAs are essential for male fertility, as shown by spermatogonial knock-out of their processing enzyme Dicer [61, 84]; the highest proportion of X-linked miRNAs is observed in mouse testis; 86% of the X-linked miRNAs escape from X inactivation during meiosis and thus might be crucial for gene expression regulation during spermatogenesis [85]. Finally, we inquired also if the targetome of X-linked miRNAs was already known to be involved in diseases using OMIM as reference database (DAVID search): five out eight statistically significant items were “diabetes”; consistently, “Insulin signaling pathway” is one of significantly enriched pathways according to KEGG. These bioinformatics results should be experimentally investigated, taking into account that diabetes is seen with four to sixfold increased frequency in Turner syndrome and Klinefelter syndrome adult patients [75, 86].

Overall, focusing the research on miRNA contribution to Turner syndrome and Klinefelter syndrome phenotype would take the understanding of syndrome development and associated morbidities to a new level, likely also in terms of therapeutic approach. For example, systematic studies on a correlation between the absence or extra-copies of specific X regions containing miRNAs, with consequent dysregulation of their potential targets, and specific clinical features should be performed. Furthermore, miRNA profiling on different tissues other than blood cells could prompt functional analysis on those miRNAs differentially expressed between X-aneuploidy and normal individuals. These studies could also pave the way for the development of new therapeutic approaches (e.g., co-administration of GH or sex hormone with an “essential” synthetic miRNA in TS).

## Conclusion and perspectives

Females appear to be equipped with a larger miRNA machinery than males: X chromosome contains an unexpected high number of miRNAs (118), in comparison to Y chromosome (4 miRNA sequences predicted) and an average of 40–50 localized on autosomes. Taking into consideration the regulatory power of these small non-coding RNAs, the scenario depicted above suggests a strong miRNA involvement in sex-specific phenotypes and some differences in pathogenetic mechanisms and/or pathological responses. This is also supported by the fact that also at cellular level, differences in response to stressful stimuli have been observed between cells carrying XX or XY chromosomes, with a possible direct role of miRNAs; as an example, miR-548am has been shown to act as a key modulator of sex difference in the susceptibility to mitochondria-based apoptosis in primary dermal fibroblasts [29]. However, most of the miRNA-based studies ignore the gender context. Some sex-biased expression of specific miRNAs could account for sex different pathophysiological conditions. This research field should be explored in the next future, to fill the gap between clinical data and our understanding of molecular mechanisms underlying some gender-biased diseases, also in terms of possible new therapeutic approaches. Noteworthy, to date most of the X-linked miRNAs have no described functions. In addition, in this review, only X-linked validated miRNAs have been considered, but they represent approximately the half of miRNA content of X chromosome; all the other miRNAs should be validated to have a comprehensive picture of their contribution to X chromosome properties and implication in the pathogenesis of X aneuploidy syndromes.

### Electronic supplementary material

Below is the link to the electronic supplementary material.

Supplementary material 1 (XLSX 129 kb)
